# Technetium-Radiolabeled Mannose-Functionalized Gold Nanoparticles as Nanoprobes for Sentinel Lymph Node Detection

**DOI:** 10.3390/molecules25081982

**Published:** 2020-04-23

**Authors:** Oscar J. Estudiante-Mariquez, Andrés Rodríguez-Galván, David Ramírez-Hernández, Flavio F. Contreras-Torres, Luis A. Medina

**Affiliations:** 1 Unidad de Investigación Biomédica en Cáncer INCan-UNAM, Instituto Nacional de Cancerología, Cd. México 14080, Mexico; ojem96@gmail.com (O.J.E.-M.); 350davidramirez@gmail.com (D.R.-H.); 2 Facultad de Química, Universidad Nacional Autónoma de México, Coyoacán, Cd. México 04510, Mexico; 3 Carrera de Biología, Unidad de Biomedicina, Facultad de Estudios Superiores Iztacala, Universidad Nacional Autónoma de México, Tlalnepantla, Estado de México 54090, Mexico; 4 Escuela de Ingeniería y Ciencias, Tecnologico de Monterrey, Nuevo León 64849, Mexico; contreras.flavio@itesm.mx; 5 Instituto de Física, Universidad Nacional Autónoma de México, Coyoacán, Cd. México 04510, Mexico

**Keywords:** gold nanoparticles, mannose, SPECT, sentinel lymph node, ^99m^Tc

## Abstract

Gold nanoparticles (AuNPs) are considered valuable nanomaterials for the design of radiolabeled nanoprobes for single-photon emission computed tomography (SPECT) imaging. Radiolabeled and functionalized AuNPs could improve lymphatic mapping by enhancing the radioactive signaling of individual particles in the sentinel node. In this study, an alternative method for functionalizing commercial AuNps with mannose is described. The chemical derivatization and biofunctionalization of AuNPs were performed with lipoic acid and mannose, respectively. Several levels of mannose were tested; the thiolate hydrazinonicotinamide-glycine-glycine-cysteine (HYNIC) molecule was also used for ^99m^Tc radiolabeling. Physicochemical characterization of this system includes U-V spectroscopy, dynamic light scattering, Fourier-transform infrared spectroscopy, and transmission electron microscopy. The most stable nanoprobe, in terms of the aggregation, radiolabeling efficiency, and purity, was tested in a sentinel lymph node model in a rat by microSPECT/computed tomography (CT) imaging. The SPECT images revealed that ^99m^Tc-radiolabeled AuNPs functionalized with mannose can track and accumulate in lymph nodes in a similar way to the commercial ^99m^Tc-Sulfur colloid, commonly used in clinical practice for sentinel lymph node detection. These promising results support the idea that ^99m^Tc-AuNPs-mannose could be used as a SPECT contrast agent for lymphatic mapping.

## 1. Introduction

Gold nanoparticles (AuNPs) have unique physical and chemical properties that make them valuable nanomaterials for many clinical applications, including their use as contrast media in diagnostic imaging [1]. The binding affinity of sulfur-based groups to gold surfaces facilitates the derivatization and functionalization of AuNPs with small molecules and biomolecules, allowing the manufacture of contrast agent systems with active targeting capabilities for multimodal imaging [2]. 

Functionalized AuNPs are ideal contrast agents for hybrid imaging techniques, for instance, single-photon emission computed tomography (SPECT) with computed tomography (CT) [3]. Radiolabeled AuNPs have been widely explored with SPECT imaging due to its high sensitivity, unlimited tissue penetration, and clinic translational capability. For example, ^125^I, ^111^In, ^131^I, and ^99m^Tc radionuclides have been attached to functionalized AuNPs [4,5,6], while the dual deposition of radionuclides (e.g., ^125^I and ^111^In) has been used to prepare multimodal probes for SPECT bioimaging [7]. In particular, SPECT/CT mapping has demonstrated a high potential to improve preoperative sentinel lymph node (SLN) localization and the reduction of false negatives compared to either CT or ultrasound scans [8,9]. The SLN denotes the first node in the lymphatic chain draining a primary tumor and provides critical diagnostic and prognostic information for patients, since it can help to identify those with signs of metastasis. In fact, there is a growing interest in the development of new and improved SPECT contrast agents for SLN detection [10].

New contrast agents should improve SLN imaging by enhancing the radioactive signaling of individual particles in the tracking of the lymphatic net and/or increase the radiotracer delivery and retention in the sentinel node [10]. Functionalized nanoparticles are relevant in SLN imaging and targeting not only because these particles can be synthesized at well-defined sizes, but also because different radionuclides can be attached to their surface, which is prepared with ligands for the active targeting of structures located at the SLN. Precise localization of unclear lymphatic nodes requires radiolabeled contrast agents exhibiting size range variation between 20 and 50 nm to be easily removed from the injection site, transported within the lymphatic network, and retained for many hours in the nodes [11]. For the efficient imaging of lymphatic tracking, the ^99m^Tc is an ideal radionuclide because of its short 6-h half-life and photon energy of 140 keV, as well as its commercial availability and low cost [11,12].

In a previous study, a multifunctional system of ^99m^Tc-labeled gold nanoparticles conjugated to HYNIC-GGC/mannose was prepared to evaluate its biological behavior as a potential radiopharmaceutical for sentinel lymph node detection in a rat model [13]. It was concluded that ^99m^Tc-AuNP-mannose remains within the first lymph node (the popliteal node) of Wistar rats for 24 h, showing the potential of this radioconjugate for the specific targeting of SLN. In the current study, an alternative method for functionalizing commercial AuNPs with mannose (AuNPs-MAN) using lipoic acid is reported. This AuNPs-MAN system was also radiolabeled with ^99m^Tc to evaluate its potential for lymph node mapping using microSPECT/CT imaging in a rat model. Physicochemical characterization of this system includes U-V spectroscopy, dynamic light scattering, Fourier-transform infrared spectroscopy, and transmission electron microscopy. 

## 2. Results

### 2.1. Preparation and Characterization of ^99m^Tc-AuNPs-MAN 

Table 1 shows the Z-potential value and hydrodynamic diameter of AuNP conjugates. The incubation of AuNPs at different times with lipoic acid (ALA) showed no significant changes. The conjugation with 2-aminoethyl 2,3,4,6-tetra-*O*-acetyl-α-d-mannopyranoside hydrochloride (MAN) or hydrazinonicotinamide-glycine-glycine-cysteine (HYNIC) did not modify the Z-potential value. Previous studies have shown that AuNPs functionalized with carboxylic acids exhibited negative Z-potential values close to −60 mV [14], suggesting that ALA can be attached to gold nanoparticles via the dithiol ring, with the carboxylic acid extending out into solution. A successful covalent functionalization between –NH_2_ (MAN) and –COOH (ALA) moieties would lead to the formation of amide groups. 

Increments in the hydrodynamic diameter were also not statistically significant; in fact, the Polydispersity Index was greater than 0.2 in all groups, indicating the presence of different size populations. It is well-known that the Dynamic Light Scattering (DLS) method gives an estimation of the hydrodynamic diameter of the particle, but it can be greatly affected by the particle shape and structure. In addition, a small portion of large contaminant or aggregated particles can greatly contribute to scattering; it is possible that the inclusion of ALA, MAN, and HYNIC attached to the surface of AuNps affects the estimation of the hydrodynamic diameter, giving information on the AuNp core, along with the coating and the layer of solvent particles attached to its surface [15]. These observations are consistent with the amount of ALA molecules chemisorbed on the surface of AuNP nanoparticles.

**Figure 1:**
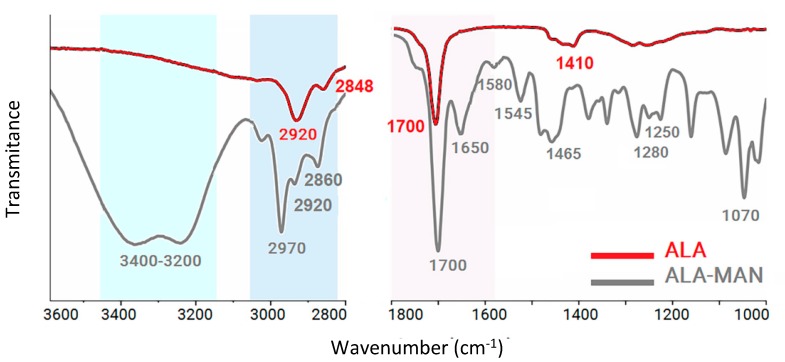
The attenuated total reflection Fourier transform infrared (ATR-FTIR) spectra for the covalent bonding of 2-aminoethyl 2,3,4,6-tetra-*O*-acetyl-α-d-mannopyranoside hydrochloride (MAN) molecules on lipoic acid (ALA) moieties.

Figure 1 illustrates the attenuated total reflection Fourier transform infrared (FTIR-ATR) studies for the covalent bonding of MAN molecules on ALA moieties. The AuNPs-ALA_48_ spectra (red line) show a peak at 1700 cm^−1^ that was assigned to C=O double bond stretching. The lipid hydrocarbon chains contribute peaks at about 2848 cm^−1^ (CH_2_, symmetric stretching) and 2920 cm^−1^ (CH_2_, asymmetric stretching). After the covalent bond (gray line), AuNPs-MAN_48_ shows a peak at ∼3400 that was assigned to O-H stretching. The peak at 2970 cm^−1^ was assigned to C-H stretching of the pyranosic sugar rings, while the peak at 1650 cm^−1^ was assigned to the stretching band of C=O stretching and C-N stretching. Furthermore, the 1580 cm^−1^ peak was associated with in-plane N-H bending and C-N stretching, while the peak at 1280 cm^−1^ was related to C-N stretching. Finally, the peak at 1070 cm^−1^ was related to C-O stretching [16].

**Figure 2:**
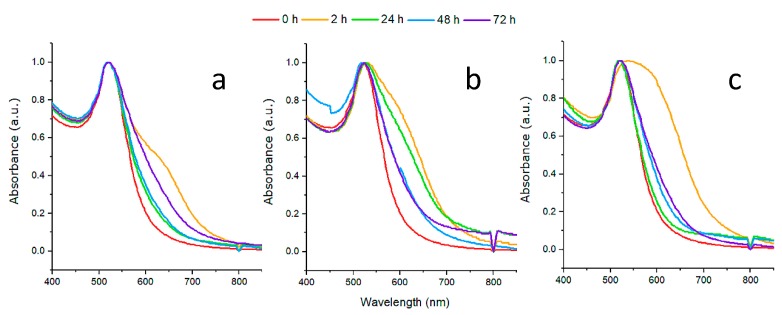
Ultraviolet-visible spectra for: (**a**) Gold nanoparticles (AuNPs)-ALA, (**b**) AuNPs-MAN, and (**c**) hydrazinonicotinamide-glycine-glycine-cysteine (HYNIC)-AuNPs-MAN. The red color indicates the spectra of pristine gold nanoparticles and the yellow, green, blue, and purple colors indicate the incubation times at 2, 24, 48, and 72 h, respectively.

The UV-Vis spectroscopy analysis of AuNPs-ALA, AuNPs-MAN, and HYNIC-AuNPs-MAN conjugates is presented in Figure 2. The AuNPs have an extinction maximum peak (λ_max_) at about 521 nm. Although the localized surface plasmon resonance (LSPR) spectrum is very sensitive to changes in the nanoparticle’s shape, size, stability, surrounding medium, and/or surface modifications [14], the location of the maximum peak does not change significantly after conjugation with ALA (Figure 2a). However, some broadening was observed for the 2 and 24 h conjugates after the covalent functionalization with MAN (Figure 2b). After conjugation with HYNIC, only the 2 h conjugate maintained the shift broadening (Figure 2c). The UV-Vis analysis indicates that in terms of aggregation, the conjugate with 48 h incubation is the most stable system.

**Figure 3:**
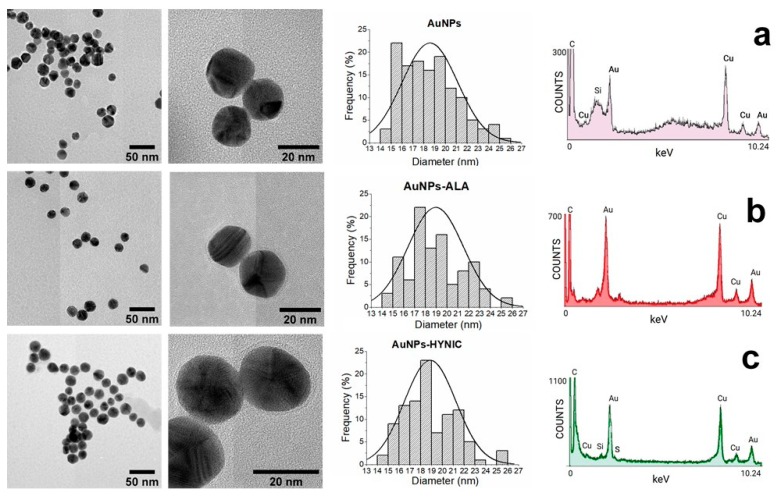
Representative transmission electron microscopy images, size histogram distribution, and HRTEM analysis for (**a**) AuNPs, (**b**) AuNPs-ALA, and (**c**) AuNPs-MAN. Results correspond to the 48 h incubation group.

Figure 3 shows the transmission electron microscopy images and HRTEM analysis. It was observed that the conjugates were spherical and no evident changes in the average particle size occurred (see the histograms of the size distribution). The average size of pristine AuNPs was 18.57 ± 2.55 nm and the size after functionalization with ALA and HYNIC was 18.95 ± 2.54 and 18.87 ± 2.37, respectively; no statistical differences were observed. A halo of low electron density material was observed around the functionalized gold nanoparticle, which was related to the presence of an ALA or MAN coating. The HRTEM elemental analysis confirmed the metallic nature of nanoparticles, as well as the presence of N and S elements in the organic molecules related to ALA and MAN.

### 2.2. Radiolabeling Analysis

According to the DLS, Z-potential and UV-Vis results, the AuNPs-MAN_48h_ and AuNPs-MAN_72h_ conjugates were selected for ^99m^Tc radiolabeling. Table 2 shows the results for the radiolabeling efficiency and stability, and the radiochemical purity. The radiochemical purity is important in radiopharmacy and SPECT imaging since it is the radiochemical form that determines the pharmacokinetics and biodistribution of the radiopharmaceutical. Given these results, AuNPs-MAN_48h_ was used for the in vivo SPECT/CT imaging study. 

### 2.3. In Vivo SPECT/CT Imaging

**Figure 4:**
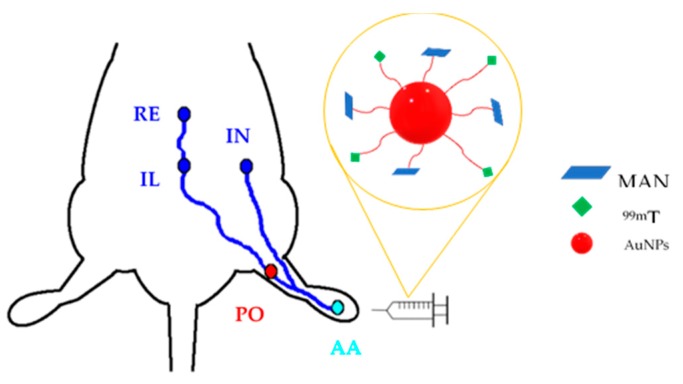
Illustrative picture of the ^99m^Tc-AuNPs-MAN_48h_ mapping in lymphatic vessels and nodes in a rat. The lymph nodes are identified as popliteal (PO), iliac (IL), inguinal (IN), and renal (RE).

Figure 4 depicts the procedure used to evaluate the lymphatic mapping of the ^99m^Tc-AuNPs-MAN_48h_. After an injection in the footpad, the contrast agent drained from the administration site (AA) into the first lymphatic node, i.e., the popliteal lymph node (PO). Next, the contrast followed the lymphatic drainage toward the iliac lymph node (IL) and then the renal node (RE). During the injection of the contrast agents, it was noticed that the ^99m^Tc-Sulfur colloid induced a significant irritation (observed as a pain reaction in the footpad during and after inoculation); this situation was not observed with the conjugated gold nanoparticles.

**Figure 5:**
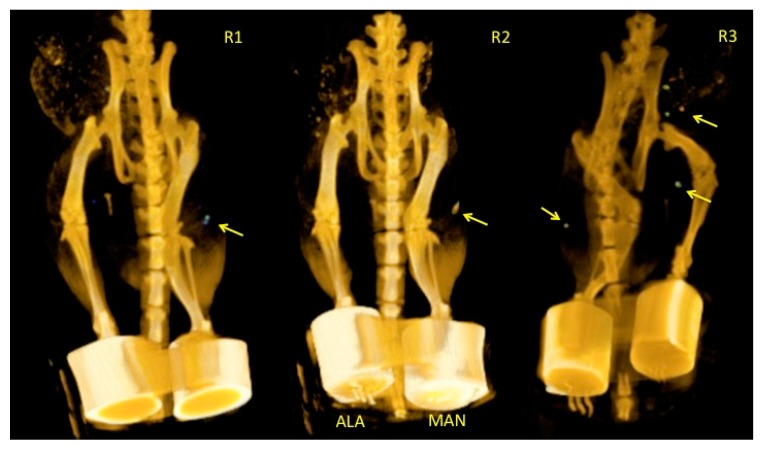
Micro single-photon emission computed tomography (SPECT)/computed tomography (CT) images depicting AuNPs-MAN_48h_ vs. AuNPs-ALA_48h_ accumulation in the popliteal lymph node. Arrows indicate the location of the nodes. ALA and MAN notation indicates the injection of AuNPs-ALA_48h_ and AuNPs-MAN_48h_ in the left and right footpad, respectively. Animals were oriented in a prone position.

Figure 5 shows representative 3D microSPECT/CT images for Group A. Here, ^99m^Tc-AuNPs-MAN_48h_ was injected in the right footpad, while ^99m^Tc-AuNPs-ALA_48h_ was injected in the left one. Lead covers were placed at the footpads to block the signal from the injection site. The images depict a preferential accumulation of AuNPs-MAN_48h_ in the PO node 3 h post injection. A minimal accumulation of AuNPs-ALA_48h_ was observed in the left PO node, except for the third rat (R3 in the figure). In this rat, accumulation in the IL and right IN nodes was also noticed. These results demonstrate mannose’s ability to promote gold nanoparticle retention within the lymph nodes.

Table 3 depicts the average percentage of the injected dose of AuNPs-MAN_48h_ and AuNPs-ALA_48h_ that stays at the injection site and the popliteal nodes. Values were calculated from the in vivo images and the ex vivo radioactivity quantification after animal sacrifice. Differences between in vivo and ex vivo results were not significant. 

**Figure 6:**
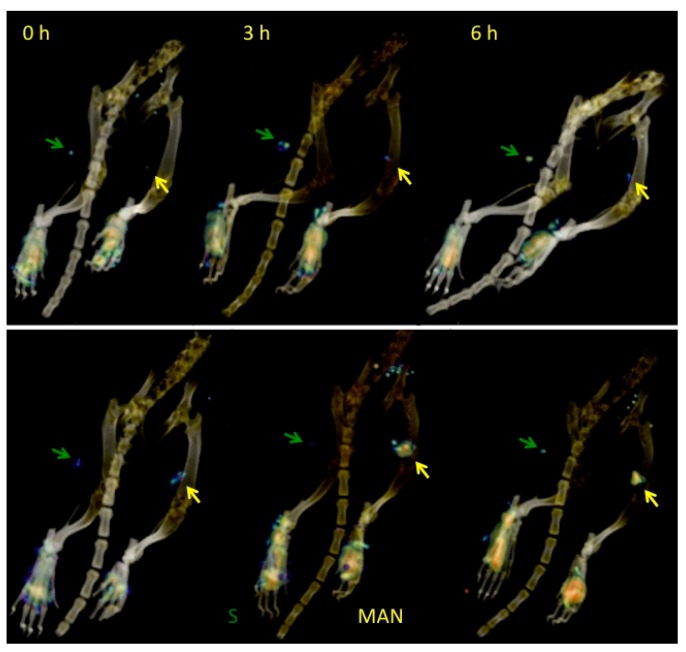
MicroSPECT/CT images of two different rats at different times, illustrating the differences in the lymphatic mapping of ^99m^Tc-Sulfur colloid (S) and ^99m^Tc-AuNPs-MAN_48h_ (MAN). Arrows indicate the location of the lymph nodes. Animals were oriented in a prone position.

Figure 6 shows representative 3D microSPECT/CT images from Group B, illustrating the lymphatic mapping sequence in two different rats at 0, 3, and 6 h after injection of ^99m^Tc-Sulfur colloid (left footpad) and ^99m^Tc-AuNPs-MAN_48h_ (right footpad). The accumulation of both contrast agents in the popliteal nodes could be observed from the beginning of the study; both contrast agents were retained in the popliteal nodes in different proportions and some moved to the next lymph nodes. Similar behavior was observed in the rest of the rats. This temporal retention of the contrast agent in the lymph nodes means that it can be employed in “radioactive staining” in order to improve SLN detection and identification. 

**Figure 7:**
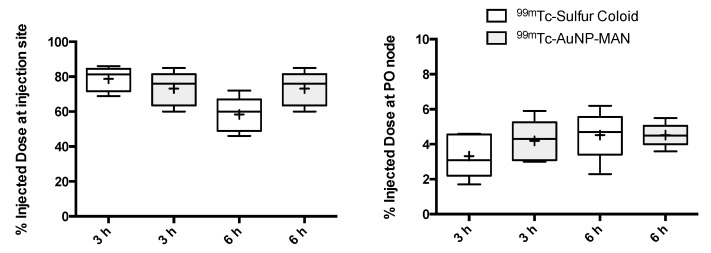
A box and whisker plot showing the results of the percentage of injected dose at the injection site (left) and popliteal node (right) at 3 and 6 h after the injection of either ^99m^Tc-Sulfur colloid or ^99m^Tc-AuNPs-MAN_48h_. Boxes extend from 1st to 3rd quartiles; thick lines and + symbols inside boxes represent the median and mean (n = 5), respectively; whiskers go from the smallest to the largest values in each group and there were no outliers. No statistical difference was observed after ANOVA.

A quantitative image analysis is presented in Figure 7; this figure describes variations as a function of time in terms of the percentage of injected dose at the injection site and popliteal lymph node. A box and whisker plot was used to illustrate the variability observed in the lymphatic mapping of each rat after the injection of both contrast media. The analysis indicates that the median and mean are almost equal. Certainly, the sample size is small, with n = 5 (one rat was not used because the images showed a dissimilar distribution of the contrast agents, related to a bad injection procedure), but it exposes the tendency of data. No statistical differences were observed, indicating a similar behavior in the lymphatic mapping and node targeting of both contrast media. The results indicate that both contrast media moved to the next nodes (the IL and IN nodes (Figure 5)). However, the percentage of injected dose accumulated in the popliteal node (median values of 3.1–4.3 and 4.5–4.7, at 3 and 6 h, respectively) allowed their detection and identification. 

## 3. Discussion

There is current interest in developing new contrast media for sentinel lymph node mapping [17,18]. In cancer therapy, the lymph node status is a good indicator of the aggressiveness of neoplasm diseases, being the factor with the greatest prognostic importance and of fundamental consideration in treatment [19]. The sentinel lymph node (SLN) is the closest node that receives lymph from the primary tumor and is the most likely place for individual cancer cells or micrometastases to be located. The presence of cancer cells in the lymph node (or nodes) is interpreted as the presence of metastasis in the patient [18]. SLN biopsy is a highly reliable method for determining the stage of the tumor, and it also helps to calculate the risk of tumor cells spreading through the lymphatic system and to plan the most appropriate treatment (radiotherapy and/or dissection). Current SLN mapping methods are intraoperative; they need to use blue dye and/or a nanometer-sized radioactive colloid injection in order to perform a visual and radioactive detection of SLN. This method is limited by the rapid movement of blue dye and the low spatial resolution and sensitivity of radiation counters in detecting radioactive colloids [20]. One of the great advantages of the timely detection of SLN is that it can help prevent more extensive lymph node surgeries. Desirable characteristics for the new contrast media includes specificity towards molecular or cellular components that characterize the lymph nodes, multifunctionality to be able to add components for imaging and/or therapy, and a cost that competes with the contrast media currently used in the clinic [6,10,13].

In the current work, we have evaluated a simple method for functionalizing commercial AuNps (~20 nm) with mannose in order to be used in SLN detection. The HRTEM, spectroscopy, and DLS techniques implemented in this study have demonstrated that AuNPs can be successfully functionalized with ALA, MAN, and HYNIC. The TEM results have shown that the average core size of gold nanoparticles does not change throughout the functionalization process (Figure 3); DLS sizing of AuNPs-MAN_48_ results in hydrodynamic diameters (i.e., the size that indicates how the particle behaves in a fluid) in the rage of 50–70 nm (Table 1). These sizes are similar in range to the current ^99m^Tc-labeled colloids employed for SLN detection: Nanocoll (albumin colloid, size ranging from 80 to 200 nm.), Nanocis (colloidal rhenium sulphide, size range of 8–68 nm); and Hepatate (tin colloid, size range of 33–255 nm) [21]. The most stable nanoprobes, in terms of their physicochemical characteristics and radiochemical’s purity and stability, were further radiolabeled with ^99m^Tc and successfully tested for lymph node mapping by microSPECT/CT in a rat model (Figure 5 and Figure 6).

To date, there are no commercial contrast agents with an optimal particle range size; however, there is a consensus that the contrast should be small enough to be rapidly removed from the injection site and transported to lymphatic nodes, and yet large enough to be retained for many hours in lymph nodes [22]. Small particles of less than 20 nm are usually cleared from the injection site and exchanged through blood capillaries. Particles of around 50 and 200 nm travel across the lymphatic capillaries and are trapped in the first lymph node [8]; large particles of hundreds of nanometers are trapped in the interstitial space and can be retained for long periods. Therefore, the hydrodynamic size of the AuNPs-MAN_48_ nanoprobes is the standard size of commercial colloids used in SLN detection. 

As was shown with the SPECT/CT studies, the conjugate ^99m^Tc-AuNPs-MAN_48_ stained the popliteal lymph node (i.e., the first draining lymph node in our experimental model) in a similar way to commercial ^99m^Tc-Sulfur colloid, which is commonly used in clinical practice. This result partially supports the potential use of ^99m^Tc-AuNPs-MAN_48_ in lymphatic mapping. A significant difference observed between both agents was the minimal irritation induced by AuNPs-MAN_48_ at the injection site compared with the sulfur colloid, which is a common side effect reported in the clinic. Another advantage of mannose-functionalized AuNPs is their potential use for multimodal photoacoustic SLN mapping, where binding to specific macrophage receptors is needed in order improve the specificity of the method [23,24]. Here, the use of mannose molecules for active targeting of the lymph node could increase the high affinity of the macrophage mannose receptor (MR, CD206), which is a C-type lectin predominantly expressed by most tissue macrophages, dendritic cells, and specific lymphatic or endothelial cells [25]. This receptor is found in lymphoid tissue at a high density and recognizes and binds to the mannose’s carbohydrate side chains [2,3]. Because the AuNPs-MAN contains a high number of mannose molecules, it shows a multivalence effect that promotes a high binding affinity (avidity) for the mannose receptor [4,26].

Finally, a slow rate of movement from the injection site was noticed for both contrast agents (Figure 3). It is important to mention that the rat’s footpads were massaged for a short period of time (1 min) after the injection, resulting in a poor stimulation for promoting the draining of the agents into the lymphatic track. It is possible that longer massaging times could result in better draining from the injection site, promoting fast accumulation of the contrast agents in the lymph nodes. However, slow draining could also permit the injection site to become a contrast depot, allowing continuous irrigation into the lymph nodes for a longer period of time.

## 4. Conclusions

In this study, we have reported a simple but efficient method for elaborating a radioactive contrast agent (^99m^Tc-AuNPs-MAN) with a high potential for lymphatic mapping using SPECT/CT imaging. In vivo studies have shown that this system can track and accumulate in lymph nodes in a similar way to the commercial ^99m^Tc-Sulfur colloid, which is commonly used in clinical practice for SLN detection. These results support the promising use of ^99m^Tc-AuNPs-MAN in SLN detection; however, more studies are required to evaluate the AuNPs-MAN´s toxicity, biodistribution, and pharmacokinetics before validating its potential clinical use for sentinel lymph node detection and targeting in cancer treatment. 

## 5. Materials and Methods 

### 5.1. Materials

Gold nanoparticles (AuNPs) with a 20 nm diameter (stabilized suspension in citrate buffer); lipoic acid (ALA), *N*-(3-dimethylaminopropyl)-*N*′-ethylcarbodiimide hydrochloride (EDC), sodium hydroxide (NaOH), ethylenediamine-*N*,*N*′-diacetic acid (EDDA), tricine, tin(II) chloride (SnCl_2_), and hydrochloric acid (HCl) were purchased from Sigma-Aldrich (Mexico City, Mexico). 2-aminoethyl 2,3,4,6-tetra-*O*-acetyl-α-d-mannopyranoside hydrochloride (MAN) was purchased from Synthose Inc, (Ontario, Canada). The modified peptide hydrazinonicotinamide-glycine-glycine-cysteine (HYNIC) was acquired from Peptides International (Kentucky, US). The ^99m^Tc pertechnetate and ^99m^Tc-Sulfur colloid were purchased from Instituto Nacional de Investigaciones Nucleares (Mexico, Mexico). All reagents were used as received, without further purification. Deionized water (18.2 MΩ cm) was used in the experiments. 

### 5.2. Preparation of ^99m^Tc-AuNPs-MAN

#### 5.2.1. AuNPs-ALA

Pristine AuNPs (3 mL, 1 nM) in sodium citrate were washed three times (11,500 rpm, 30 min) and resuspended in deionized water at pH 11 (NaOH, 1 M). Then, 2.5 mL of AuNPs (1 nM) was incubated with 250 µL of ALA (10 mM) for 2, 24, 48, or 72 h at room temperature and constant stirring of 3000 rpm [25]. For each time of incubation, free ALA was removed by ultrafiltration (Ultrafree-PF filters 10,000 NWWL, Millipore); the AuNPs-ALA pellets were washed three times (11,500 rpm, 30 min) and resuspended in deionized water at pH 7 and adjusted to a nanoparticle concentration of 1 nM using Equation (1) [27]:

*c* = *A*_450_/*Ɛ*_450_ (1)

where *A*_450_ is the absorption of gold nanoparticles at 450 nm for a standard path length *l* of 1 cm and *Ɛ*_450_ is the molar decadic extinction coefficient at λ = 450 nm for 20 nm gold nanoparticles. These conjugates were labeled according the incubation time, AuNPs-ALA_(h)_, (where, h = 2, 24, 48, or 72 h).

The number of ALA molecules bonded to AuNPs was estimated by a UV-vis calibration curve of ALA concentrations (from 0.5 to 1.5 mM, plus a blank sample). The specific absorption peak at 330 nm of the five-membered ring in lipoic acid [28] was selected for the quantification. A UV-vis spectrophotometer (Beckman Coulter, California, US) was used to obtain the absorption spectrum as a function of the number of ALA molecules at each molar concentration. From a linear fit of data, the number of remaining free ALA molecules in solution after centrifugation was calculated; these values were used to estimate the number of ALA molecules per AuNp: 1107± 587 at 2 h, 4151 ± 4381 at 24 h, 4428 ± 479 at 48 h, and 5535 ± 2089 at 72 h. The number of nanoparticles was calculated using equation (2):

*N* = *M_C/m_* (2)

where *M_C_* is the mass concentration of gold (g/mL) and *m* is the mass of an individual nanoparticle (g/particle).

#### 5.2.2. AuNPs-MAN

One milliliter of each AuNPs-ALA_(h)_ (1 nM, pH 7) was mixed with 2 μL of MAN (100 mg/mL in ethanol) and 10 µL of EDC (40 mM, pH 6.5). These conjugates were labeled as AuNPs-MAN_(h)_ (h = 2, 24, 48, or 72 h). The solutions were incubated with constant stirring (3000 rpm, 5 h), centrifuged at 11,500 rpm (30 min), washed three times in deionized water at pH 11, and left in basic hydrolysis at pH 11 for 24 h; finally, AuNPs-MAN_(h)_ conjugates were adjusted to a nanoparticle concentration of 1 nM. 

#### 5.2.3. HYNIC-AuNPs-MAN 

One milliliter of each AuNPs-MAN_(h)_ (1 nM) was incubated with HYNIC (5 µL, 1 mM in ethanol) with constant stirring (3000 rpm, 20 min). The conjugates were labeled as HYNIC-AuNPs-MAN_(h)_ (h = 2, 24, 48, or 72 h). Unbound HYNIC was removed by ultrafiltration (Ultrafree-PF filters 10,000 NWWL, Millipore). The nanoparticles were washed three times (11,500 rpm, 30 min), resuspended in deionized water at pH 11, and adjusted to a nanoparticle concentration of 1 nM.

The number of HYNIC molecules per AuNP was also estimated by a UV-vis calibration curve from HYNIC (from 2 to 7 μM, plus a blank sample). The absorbance peak at 254 nm was used [29]; the absorption spectrum was plotted as a function of the number of HYNIC molecules at each molar concentration. The parameters from the linear fit of data were used to calculate the remaining free HYNIC molecules in solution after centrifugation; then, the number of HYNIC molecules per nanoparticle was estimated as 1199 ± 430 at 20 min, 2214 ± 146 at 2 h, 3063 ± 360 at 24 h, and 2989 ± 387 at 48 h. The number of nanoparticles was calculated using Equation (2).

#### 5.2.4. Characterization

Absorption spectra (400–1000 nm) for each AuNPs-MAN_(h)_ were obtained with a Beckman Coulter DU-530 Life science UV/Vis spectrophotometer using a 1 cm quartz cuvette. UV/Vis analysis was used to monitor the AuNP surface plasmon band (520 nm) shift in order to evaluate the conjugation stability. 

The hydrodynamic diameter and Z-potential were measured by Dynamic Light Scattering (DLS) and Electrophoretic Light Scattering, respectively, using a Z-sizer 90Plus Analyzer (Brookhaven Instruments Corporation, Long Island, NY, USA). In this equipment, the default calculation employs the Smoluchowski limit to calculate the Z-potential and the Stokes–Einstein equation for particle sizing. Smoluchowski approximation is valid for nanoparticles in aqueous media, even though the smallest nanoparticles (<20 nm diameter) may not have κa >> 1 necessary to justify the application of Smoluchowski approximation [30]. κa >> 1 indicates that the particle radius (a) is large compared to the Debye length (1/κ) (1/κ is ~10 nm for 1 mM aqueous salt solutions), which is used in Henry’s function (F(κa)) to calculate the electrophoretic mobility using Henry’s equation [30]. In this work, samples (~20 nm AuNps) were analyzed in deionized water and adjusted to pH 7 with 1 M NaOH at 25 °C, and the final concentration was 1 mM of NaOH. Polystyrene nanospheres (100 nm) (Duke Scientific, California, US) and BI-ZR3 zeta potential reference material (Brookhaven Instruments Corp., New York, US) were used as standards to verify the equipment performance before measurements. 

The attenuated total reflection Fourier transform infrared (FTIR-ATR) spectra, from 1000 to 3800 cm^−1^, of the covalent reaction for ALA-MAN, were recorded on a Perkin-Elmer Spectrum 100 spectrometer (Perkin Elmer, Ohio, US) to corroborate the formation of amide I and II bonds by comparing the spectra of AuNPs-ALA_48_ before and after its conjugation with MAN. Samples were deposited on the ATR crystal by the drop casting technique and deposition was repeated several times.

Morphology and chemical composition analysis were performed using a JEM-2010F FASTEM instrument (JEOL, Massachusetts, US) coupled to a NORAN energy dispersive spectrophotometer (EDS) operating at 20 kV. Samples were prepared by applying 30 μL of a diluted nanoparticle solution onto carbon-coated copper grids (Ted Pella, California, US). Excess solution was removed with filter paper and the sample was allowed to dry at room temperature overnight. The resulting images were analyzed using ImageJ version 1.40 software (NIH, Wayne Rasband); for size analysis, at least 100 nanoparticles were evaluated per sample and the statistical analysis was performed with OriginPro2020 software (Northampton, US).

#### 5.2.5. Radiolabeling

Radiolabeling was performed as previously reported [13]. Briefly, 0.5 mL of ^99m^Tc pertechnetate (1 mCi) with 60 μL of SnCl2 (10 mM, pH 1) and 2 µL of HCl (12 M) were incubated at 30 °C under constant stirring (500 rpm, 15 min). Then, the pH was adjusted to 7 (NaOH, 1 M) for further adding 40 µL of tricine (30 mM, 0.1 M phosphate buffer, pH 7.4) at 30 °C under constant stirring (500 rpm, 10 min). Finally, 1 mL of each HYNIC-AuNPs-MAN_(h)_ (1 nM) and 10 μL of EDDA (20 mM, 0.1 M phosphate buffer, pH 7.4) were added to the previous solution and incubated for 20 min at 100 °C under stirring at 500 rpm. Purification was performed by filtration using Amicon centrifugal filters (0.5 mL ultracel, 100 K) at 12,000 rpm, for 5 min. Radiolabeled nanoparticles (^99m^Tc-AuNPs-MAN_(h)_) remained in the filter, while free pertechnetate (^99m^TcO_4_^−^) and hydrolyzed/reduced technetium (^99m^TcO_2_) passed through the filter. The radiolabeling efficiency was evaluated by measuring the radioactivity (mCi) in the supernatant and pellet, using a dose calibrator (34-056 Deluxe Isotope Calibrator II, Nuclear Associates).

The radiochemical purity (*RP*) was determined by ITLC-SG (General Electric, Santa Clara, CA, USA). The samples (2 μL) were spotted on ITLC strips and 2-butanone and saline solution (NaCl-0.9%) were used as mobile phases. The 2-butanone was used to evaluate the percentage of free ^99m^TcO_4_^−^ and the saline solution was employed to measure the percentage of hydrolyzed/reduced technetium (^99m^TcO_2_). The strips were cut in half and the radioactivity in each segment was measured using a well-type gamma counter (Ludlum 2200, Sweetwater, TX, USA). The *% RP* was calculated as

(3) $$\%RP=\frac{B}{B+T}\times 100$$

where *B* and *T* represent the radioactivity (cps) measured at the bottom (i.e., ^99m^Tc-AuNPs-MAN) and top segments (i.e., ^99m^TcO_4_^−^ or ^99m^TcO_2_), respectively.

The radiolabeling stability was measured in saline solution (NaCl 0.9%) at room temperature and in fresh human serum (37 °C). A total of 100 μL of ^99m^Tc-AuNPs-MAN_(h)_ was added to 500 μL of either saline solution or human serum and incubated for 6 h. The stability, as a function of time, was determined by ITLC-SG/NaCl-0.9%, as previously described, after collecting samples (15 μL) at 15 min; 30 min; and 1, 2, 4, and 6h. All measurements were corrected by radioactivity decay.

### 5.3. Animal Model

Male Wistar rats (250–300 g) were obtained from the UNAM’s Medical School animal facility (Mexico City). Animals were kept in a pathogen-free environment and fed with autoclaved food and water ad libitum. The procedures for care and use of the animals were approved by local institutional Scientific and Ethics Committees at INCan (019/010/IBI) (CEI/1349/18) (2019/0403/CB1) and all applicable institutional and governmental regulations were followed in accordance with the Federal Regulations for Animal Production, Care and Experimentation (NOM-062-ZOO-1999, Ministry of Agriculture, Mexico, Mexico). The guidelines from the Guide for the Care and Use of Laboratory Animals of the National Institute of Health ((NIH, Washington D. C., US) were also followed. All efforts were made to minimize animal suffering and to reduce the number of animals used in the experiments.

To evaluate the potential of ^99m^Tc-AuNPs-MAN as a contrast agent for SLN mapping, the foot-draining popliteal lymph node model of rats was chosen. This model is widely used to test diagnostic techniques of metastasis and for drug delivery to lymphatic nodes. In this model, the administration of ^99m^Tc-AuNPs-MAN was performed in the footpad of Wistar rats. It is expected that ^99m^Tc-AuNPs-MAN will drain from the administration area to the first lymphatic node, i.e., the popliteal lymph node. 

### 5.4. In Vivo SPECT/CT Imaging

A microPET/SPECT/CT imaging system (Albira ARS, Bruker, Billerica, Spain) was used to analyze the in vivo lymphatic mapping of the radiolabeled conjugates in healthy rats. Nine rats were used in this study, organized into two experimental groups. In the first group (Group A), three rats were used to compare the popliteal node targeting of ^99m^Tc-AuNPs-MAN_48h_ vs. ^99m^Tc-AuNPs-ALA_48h_. This experiment was designed to verify the lymphatic node accumulation of nanoparticles coated with mannose. The other six rats (Group B) were used to evaluate the targeting efficiency of ^99m^Tc-AuNPs-MAN_48h_ vs. ^99m^Tc-Sulfur colloid. This colloid is commonly used in clinical practice for the assessment of lymphatic tracking and sentinel node targeting and was used here as a gold standard.

The radiolabeled complexes were injected subcutaneously into the footpad of rats under anesthesia using a mixture of oxygen/isofluorane at 3%. The footpad of each animal received a single injection (~50 µCi) of ^99m^Tc-AuNPs-MAN_48_, ^99m^Tc-AuNPs-ALA_48h_, or ^99m^Tc-Sulfur colloid in approximately 35 µL. After the injection, the footpad was gently massaged for one minute to promote the movement of the complex into the lymphatic pathway. For the first experiment (Group A), images were acquired at 1 and 3 h post injection and the animals were sacrificed by cervical dislocation after acquiring the 3 h image; popliteal lymph nodes and the injection site were dissected. The nodes were harvested, weighed, and counted for radioactivity using the well-type gamma counter (Ludlum Model 2200 Scaler/Ratemeter, Texas, US). For the second experiment (Group B), images were acquired at 1, 3, and 6 h post injection.

All images were reconstructed with Albira’s reconstruction software and the quantification analysis and image processing were performed with PMODE (PMODE Technologies, Ltd. Zurich, Switzerland) and Osirix MD (Pixmeo SARL), respectively.

### 5.5. Statistical Analysis

The results are expressed as the mean ± SD (standard deviation). Statistical analysis was performed using one-way analysis of variance (ANOVA). Significance was assumed at *p* < 0.05. All assays were performed with at least three independent triplicates. 

## Figures and Tables

**Table 1:** Z-potential values and hydrodynamic diameter (in parentheses) for synthesized particles. Values represent the mean value ± SD.

	2 h *	24 h *	48 h *	72 h *
AuNPs	(30 ± 1.6)	(33 ± 6.5)	(38 ± 5.6)	(31 ± 4.1)
AuNPs-ALA	−53.7 ± 16.0 (73. ± 31.3)	−63.7 ± 6.2 (73. ± 36.3)	−73.6 ± 1.4 (60.3 ± 23.2)	−64.2 ± 4.1 (59.9 ± 23.9)
AuNPs-MAN	−58.5 ± 8.1 (103.5 ± 46.4)	−65.9 ± 7.4 (82.1 ± 37.9)	−67.8 ± 10.4 (71.1 ± 18.7)	−60.25 ± 8.38 (69.8 ± 19.3)
HYNIC-AuNPs-MAN	−59.2 ± 10.8 (72.6 ± 51.6)	−59.5 ± 6.5 (76.8 ± 28.7)	−59.7 ± 7.2 (49.8 ± 14.)	−71.1 ± 10.6 (68.6 ± 29.4)

* Z-potential is presented in mV and size is presented in nm. All measurements were conducted in deionized water at pH 7.0 adjusted with NaOH (1 M).

**Table 2:** Radiolabeling results.

Conjugate	Radiolabeling Efficiency	Radiochemical Purity	Radiolabeling Stability *
^99m^Tc-AuNPs-MAN_48h_	86 ± 14%	97 ± 2%	99 ± 2.3%
^99m^Tc-AuNPs-MAN_72h_	60 ± 30%	84 ± 20%	96 ± 2.1%

* In vitro stability assay in saline solution (NaCl 0.9%) and fresh human serum for a 6 h period.

**Table 3:** Average percentage of injected dose at injection site and popliteal nodes.

	AuNPs-ALA_48h_		AuNPs-MAN_48h_	
	Injection Site	Popliteal Node	Injection Site	Popliteal Node
In vivo	74.2 ± 9.4	1.0 ± 0.3	79.3 ± 11.5	3.1 ± 2.7
Ex vivo	80.4 ± 6.9	3.4 ± 1.3	77.4 ± 5.3	3.1 ± 0.9

Values represent the average ± SD from n = 3. Values were corrected by radioactive decay.
